# The Characteristics of Psychiatrists Disciplined by Professional Colleges in Canada

**DOI:** 10.1371/journal.pone.0050558

**Published:** 2012-11-28

**Authors:** Asim Alam, Paul Kurdyak, Jason Klemensberg, Joshua Griesman, Chaim M. Bell

**Affiliations:** 1 Department of Anesthesiology, Hospital for Sick Children, Toronto, Canada; 2 Department of Laboratory Medicine & Pathobiology, University of Toronto, Toronto, Canada; 3 Department of Medicine, University of Toronto, Toronto, Canada; 4 Department of Psychiatry, University of Toronto, Toronto, Canada; 5 Institute for Health Policy Management and Evaluation, University of Toronto, Toronto, Canada; 6 Centre for Addiction and Mental Health, Mount Sinai Hospital, Toronto, Canada; 7 Mount Sinai Hospital, Toronto, Canada; 8 Royal College of Surgeons in Ireland, Toronto, Canada; The University of Queensland, Australia

## Abstract

**Background:**

The identification of health care professionals who are incompetent, impaired, exploitative or have criminal intent is important for public safety. It is unclear whether psychiatrists are more likely to commit medical misconduct offences than non-psychiatrists, and if the nature of these offences is different.

**Aim:**

The aim of this study was to compare the characteristics of psychiatrists disciplined in Canada and the nature of their offences and disciplinary sentences for the ten years from 2000 through 2009 to other physicians disciplined during that timeframe.

**Methods:**

Utilizing a retrospective cohort design, we constructed a database of all physicians disciplined by provincial licensing authorities in Canada for the ten years from 2000 through 2009. Demographic variables and information on type of misconduct violation and penalty imposed were also collected for each physician disciplined. We compared psychiatrists to non-psychiatrists for the various outcomes.

**Results:**

There were 82 (14%) psychiatrists of 606 physicians disciplined in Canada in the ten years from 2000 through 2009, double the national proportion of psychiatrists. Of those disciplined psychiatrists, 8 (9.6%) were women compared to 29% in the national cohort. A total of 5 (6%) psychiatrists committed at least two separate offenses, accounting for approximately 11% of the total violations. A higher proportion of psychiatrists were disciplined for sexual misconduct (OR 3.62 [95% Confidence Interval [CI] 2.45–5.34]), fraudulent behavior (OR 2.32 [95% CI 1.20–4.40]) and unprofessional conduct (OR 3.1 [95% CI 1.95–4.95]). As a result, psychiatrists had between 1.85–4.35 greater risk of having disciplinary penalties in almost all categories in comparison to other physicians.

**Conclusion:**

Psychiatrists differ from non-psychiatrist physicians in the prevalence and nature of medical misconduct. Efforts to decrease medical misconduct by psychiatrists need to be conducted and systematically evaluated.

## Introduction

There have been conflicting studies examining the relationship between the specialty of psychiatry and medical misconduct. Initial series found that psychiatrists were less likely to incur malpractice claims or lose malpractice insurance than other specialists in the US [1]–[3]. However, other work has shown that psychiatrists have an increased risk for disciplinary action by licensing authorities.[4]–[6] A comprehensive study on disciplinary action by the California Medical Board found that psychiatrists were significantly more likely than non-psychiatrists to be disciplined for sexual relationships with patients and about as likely as other physicians to be charged with negligence or incompetence. [7] This work concluded that psychiatry, as a profession, had an obligation to address sexual contact with patients and other causes for medical board discipline through enhanced education and changes to licensure standards.

Since these studies from the late 1990s and early 2000s, no follow up has been done to determine whether the rate of medical misconduct, and specifically sexual misconduct, has reduced amongst psychiatrists. In addition, there has been no national-level data on psychiatrists and disciplinary action, including any data on disciplined psychiatrists from Canada. Therefore, we examined the characteristics of psychiatrists in a consecutive series of physicians disciplined in Canada in the ten years from 2000 through 2009.

## Methods

In Canada, provincial legislation provides the legal basis for provincial licensing authorities known as the Colleges of Physicians and Surgeons (CPSs). These regulatory authorities provide the structure for governance, discipline, and accountability for physicians in Canada. Information regarding physician-related complaints is usually confidential unless the complaints lead to a formal disciplinary hearing. Information on disciplinary hearings and proceedings from the territorial licensing authorities (Northwest Territories, Nunavut, Yukon) are largely excluded from these rules. [8].

### Database Construction

After receiving ethics approval from the St. Michael’s Research Ethics Board, we constructed a database of all Canadian physicians disciplined in the ten years from 2000 through 2009. Detailed methodology for this retrospective cohort has been described previously. [8] Briefly, physicians were identified by reviewing all available online publications on physician discipline from each provincial CPS. Demographic information collected for each physician included: sex; type of practice license (independent practice vs educational license); medical school of graduation (domestic vs international medical graduate); and medical specialty. Specialties were grouped into two categories: 1) psychiatrists; and 2) all other physicians. We calculated total years of practice as the total number of years between obtaining a medical degree and the disciplinary action.

Additional information on physicians not available through the discipline summaries was obtained from provincial licensing website databases, the Canadian Medical Directory (between the years of 1970–2008) or the CPSs themselves. [9].

Each disciplinary action was reviewed and grouped according to modified categories: conviction of a crime; fraudulent behaviour/prevarication; inappropriate prescribing; mental illness; failure to meet a standard of care; use by the physician of drugs or alcohol; sexual misconduct; unprofessional conduct; unlicensed activity/breech of registration terms; miscellaneous violations; and unknown/unclear violations.[4]; [5]; [8] Miscellaneous violations mainly included violations involving breaches of confidentiality, improper disclosure to patients and improper handling or maintenance of medical records. The imposed penalties were grouped into the following categories: license revocation; license surrender; suspension; license restriction; mandated retraining/education/course/assessment; mandated psychological counseling and/or rehabilitation; formal reprimand; fine/cost repayment; other actions.

### Statistics

We calculated the frequencies and proportions of each physician characteristic, violation and penalty category variable. Information on total numbers of physicians (excluding resident physicians) in the years of study was obtained from the Canadian Institute of Health Information. [10] Utilizing these sources, we calculated the average number of psychiatrists and non-psychiatrists practicing in Canada between 2000–2009. Statistical analysis comparing disciplined psychiatrists to non-psychiatrists were calculated using a JavaStat (www.statpages.org) statistical computation.

## Results

In the ten years from 2000 through 2009, 606 physicians were disciplined in Canada, corresponding to a rate of about 0.06–0.11% per year. [8] Of these, 82 (14%) were psychiatrists.(Table 1). This is approximately double the national proportion of psychiatrists, which between 2000–2009, represented 7% of all physicians.(8) Of the disciplined psychiatrists, 8 (9.6%) were women; nationally, approximately 29% of all psychiatrists are women. [11] In comparing the 82 psychiatrists and 524 non-psychiatrists there were similar proportions for sex, international medical graduates, and resident trainees compared to non-psychiatrist counterparts.(Table 1) The mean (SD) number of years of practice before conviction was 33 (11) years.

**Table 1:** The characteristics of disciplined psychiatrists and other physicians in Canada for the ten years from 2000 through 2009.

		Psychiatrists Disciplinedn = 82		Non-Psychiatrists Disciplinedn = 524		p-Value
Characteristic		Frequency	%	Frequency	%	
**Sex**						
	Female	8	9.76	41	7.82	0.517
	Male	74	90.24	483	92.18	
**Licence Type**						
	Independent	81	98.78	518	98.85	1.00
	Resident Trainee	1	1.22	6	1.15	
**Location of Medical School**						
	International Medical Graduate	30	31.71	175	33.40	0.616
	Canada	52	63.41	349	66.60	

A total of 852 different offenses were committed by the 606 disciplined physicians. Of these offences, 172 (20.2%) were of a sexual nature. Psychiatrists were disciplined for 35 (20.3%) sexual misconduct offences in the ten years from 2000 through 2009, and were more likely to be disciplined for sexual misconduct than other disciplined physicians (OR 3.62 [95% confidence interval [CI] 2.45–5.34]), fraudulent behavior (OR 2.32 [95% CI 1.20–4.40] and unprofessional conduct (OR 3.1 [95% CI 1.95–4.95]) (Table 2). Of these 82 psychiatrists, 5 (6%) committed repeat offences; that is, they committed at least two different offenses at different time points in the ten years from 2000 through 2009. In this manner, repeat offenders accounted for approximately 11% of both total violations and penalties for all psychiatrists. Amongst the repeat offenders, no offender was disciplined for sexual misconduct more than once.

**Table 2:** The types of violations of disciplined psychiatrists and other physicians in Canada for the ten years from 2000 through 2009.

Types of violations	Psychiatrists Who Committed Violations (N = 82)			Non-Psychiatrists Who Committed Violations (N = 524)			Analysis
	N	Percent of Violators	Percent of All Psychiatrists*	N	Percent of Violators	Percent of All Non-Psychiatrists^†^	OR [95% CI]
**Conviction of a Crime**	5	6.1%	0.12%	29	5.5%	0.05%	2.43 [0.83–6.59]
**Fraudulent Behaviour/Prevarication**	12	14.6%	0.29%	73	13.9%	0.13%	2.32 [1.20–4.40]
**Inappropriate Prescribing**	4	4.9%	0.10%	70	13.4%	0.12%	0.80 [0.25–2.29]
**Miscellanous violations**	9	11.0%	0.22%	95	18.1%	0.16%	1.34 [0.63–2.73]
**Mental Illness**	0	0.0%	0.00%	2	0.4%	0.00%	0.00 [0.00–57.18]
**Self use of drugs and alcohol**	1	1.2%	0.02%	10	1.9%	0.02%	1.40 [0.07–10.59]
**Sexual misconduct**	35	42.7%	0.86%	137	26.1%	0.24%	3.62 [2.45–5.34]
**Standard of Care Issue**	12	14.6%	0.29%	151	28.8%	0.26%	1.21 [0.59–2.07]
**Unclear violations**	2	2.4%	0.05%	16	3.1%	0.03%	1.76 [0.28–7.96]
**Unlicensed activity**	7	8.5%	0.17%	49	9.4%	0.09%	2.02 [0.84–4.63]
**Unprofessional conduct**	24	29.3%	0.59%	109	20.8%	0.19%	3.1 [1.95–4.95]

Psychiatrists had a 1.85–4.35 times greater risk of having a disciplinary penalty than other physicians in most categories, except psychotherapy or counseling (Table 3). Specifically, psychiatrists had more 4 times the likelihood of voluntarily surrendering their license and more than 3 times the likelihood of license revocation.(Table 3). There were 8 (9.8%) psychiatrists who voluntarily surrendered their license and 16 (19.5%) who had their license revoked. Of these 16 psychiatrists, 13 (81%) were disciplined for sexual misconduct issues.

**Table 3:** Types of penalties imposed on psychiatrists and other physicians disciplined in Canada for the ten years from 2000 through 2009.

	Psychiatrists Who Committed Violations (N = 82)			Non-Psychiatrists Who Committed Violations (N = 524)			Analysis
Types of penalties imposed	N	Percent ofViolators	Percent of AllPsychiatrists*	N	Percent of Violators	Percent of All Non-Psychiatrists^†^	OR [95% CI]
**Fine/Cost**	52	63.4%	1.27%	364	69.5%	0.63%	2.03 [1.47–2.74]
**Formal Reprimand**	39	47.6%	0.95%	234	44.7%	0.41%	2.36 [1.67–3.36]
**Other Action**	7	8.5%	0.17%	26	5.0%	0.05%	3.80 [1.50–9.19]
**Psychotherapy/Counseling/Substance Abuse Program**	8	9.8%	0.20%	50	9.5%	0.09%	2.26 [0.99–4.95]
**Restriction**	25	30.5%	0.61%	157	30.0%	0.27%	2.25 [1.44–3.50]
**Retraining/Course/Assessment Required**	19	23.2%	0.47%	120	22.9%	0.21%	2.22 [1.34–3.71]
**Revocation**	16	19.5%	0.39%	73	13.9%	0.13%	3.099 [1.73–5.46]
**Voluntary Surrender (License)**	8	9.8%	0.20%	26	5.0%	0.05%	4.35 [1.81–10.05]
**Suspension**	34	41.5%	0.83%	259	49.4%	0.45%	1.85 [1.28–2.69]

## Discussion

We studied all physicians disciplined through provincial authorities in Canada for the ten years from 2000 through 2009 and compared psychiatrists with other physicians. We found that psychiatrists were disproportionally represented amongst disciplined physicians and were more likely to be disciplined for sexual misconduct, fraudulent behavior and unprofessional conduct. We also found that, similar to previous studies, women accounted for a small proportion of these cases [8]. About 6% of the disciplined psychiatrists were repeat offenders but these accounted for 11% of all disciplinary cases. Furthermore, as to be expected, psychiatrists had a much higher risk of having almost all penalties than other physicians.

In our preceding study on physician discipline in Canada, we found that physicians were disciplined at a rate much lower than that found in other jurisdictions, notably the United States (US). [8] The percentage of physicians disciplined in the US is approximately 0.5%; higher than the approximately 0.1%observed in Canada. [8] Two notable differences accounting for this phenomenon include divergent medical licensure policies and differences amongst the litigiousness of the respective populace. [8].

Since 2001, no work has specifically examined misconduct within the field of psychiatry. [7]. We have previously shown that psychiatrists were overrepresented among disciplined physicians, the second largest group of physicians disciplined next to family physicians. [8] Although family physicians represented the largest specialty of physicians in Canada, psychiatrists were far from being the second largest physician cohort. [8] The current study further demonstrates that psychiatrists are more likely to be disciplined for sexual misconduct than other types of physicians. Despite this high rate of sexual misconduct, only 16 (19.5%) psychiatrists who were disciplined had their licenses revoked. Nevertheless, more than 80% of these revocations were for sexual misconduct issues. Similar to previous findings, our study suggests that psychiatrists proportionally received more disciplinary penalties than other physicians.[4]; [6]; [7]; [12].

The increased risk of being disciplined for sexual misconduct found in our analysis has been corroborated in at least four other previous studies.[7]; [13]–[15] The persistence of serious boundary violations in the field of psychiatry has prompted evaluation of the issue. Risk factors for boundary violations within the psychiatrist/patient relationship have been grouped into therapist risk factors (e.g. life crises, transitions, therapist illness, loneliness and the impulse to confide, problems with limit setting, “small town” issues) and patient risk factors (e.g. excessive dependency on the therapist, retraumatization, and transference).[16]; [17] Very few studies have focused specifically on psychiatrists who violate boundaries. Gabbard wrote about three categories of psychotherapists who have sexually abused patients: 1) the smallest group consists of psychotic therapists whose abusive behaviour is based on delusional thoughts; 2) the next largest category are antisocial and their exploitative behaviour is observed in all their relationships, and not solely their therapeutic ones; and 3) the largest category, called the “lovesick” therapist – typically neurotic or characterologically disturbed middle-aged men who are socially isolated and who “fall in love” with female patients much younger than themselves. [18] However, a Canadian longitudinal study of psychiatrists followed from residency training has demonstrated that psychiatrists who ultimately lose their license for sexually exploitative behaviour with patients have antisocial characteristics based on personality trait testing. [19] The authors of this study suggest that personality traits that predict exploitative behaviour may be detectable when medical students apply for residency training programs, raising the interesting, and ethically challenging prospect of screening for exploitative behaviour in an effort to protect the public.

Psychiatrists may also be at a greater risk for being disciplined for drug and alcohol problems, fraud, and theft. [20], [21], [22] In our series, psychiatrists were indeed at increased risk in comparison to other physician groups for fraudulent behavior and unprofessional conduct when we examined the entire physician population, thereby corroborating previous findings. However, they were not at an increased risk for self-use of drugs and alcohol. The reasons for this are multifold and our research cannot make detailed inferences about the exact mechanism behind this phenomenon.

The observation that psychiatrists have a greater risk of receiving almost all types of disciplinary penalties is corroborated by previous work.[4]; [6]; [7]; [12] A recent series by Cardarelli et al. suggests that psychiatrists had more than two and a half times the risk for license revocation following discipline. [23] We observed that a large proportion of revocations were indeed for sexual misconduct. A further case-by-case analysis proved that some of these revocations came only after a physician refused to cooperate with initial, less severe sanctions. A paucity of repeat sexual offenses from repeat offenders may indicate this approach is indeed an appropriate measure to protect the public from these offenders once they identified. However, more research will be needed to determine the relationship between the nature of sanctions and deterrence of sexual misconduct.

This study has a number of limitations that have been outlined previously. [8] Firstly, we were unable to obtain data for disciplinary action in the territories and in certain provinces for specific years under study. However, we believe that this data would only represent few disciplined physicians as a whole, based on the proportion of physicians disciplined from these provinces. Second, we had to exclude findings where the physician’s name was not published. These physicians represented only 23 (4%) of the total disciplined physicians, a relatively small proportion. Finally, our data pertain only to disciplinary actions and do not inform the degree and nature of patient complaints. It may be that investigations, and therefore disciplinary hearings, are more likely to proceed when the patient complaint is sexual in nature.

We do not assume there is a direct relationship between discipline and misconduct; indeed, patients may be more likely to report sexual misconduct for physicians of particular specialties or for particular types of misconduct. The nature of the problems that may render psychiatric patients especially vulnerable to inappropriate caregiver relationships may also make them more reluctant or conversely, inclined, than most to report sexual misgivings. [7] We acknowledge this problem with interpretation and urge further study to resolve this ambiguity.

Prevention of boundary violations is a difficult goal. There has been a significant response to the persistent sexual misconduct observed among psychiatrists. For example, academic psychiatrists have developed curricula for psychiatric residents that address the issue of boundary violations in the therapeutic relationship [24]. Still, there is a paucity of evidence suggesting that practitioners and residents are widely being taught the central psychodynamic notions involved in evolving sexual misconduct with patients. However, psychiatric associations in a number of jurisdictions have published position statements in an effort to reduce boundary violations generally, and sexual misconduct specifically, among psychiatrists.[25]; [26] As well, there is a suggestion that mandatory consultation and supervision with colleagues in the field is a key tool in combating sexual misconduct. [27] Plakun states that professionals who “routinely present their work to others in consultation, supervision, peer discussion groups, or case presentation are probably at less risk of becoming isolated” and “lost in the dyad in their work” and this would potentially mitigate their risk of sexual misconduct. [27].

Discipline for complaints of sexual misconduct, fraud and unprofessional conduct is more likely to occur against psychiatrists than other specialties. Pointedly, the high rate of sexual misconduct relative to other specialties has persisted despite widespread efforts to prevent boundary violations among practicing psychiatrists. While the absolute rate of sexual misconduct is low, even a few clinicians who violate professional conduct boundaries can have great potential to harm patients, as well as public trust. A focus on preventing disciplinary action is a fundamental issue of patient safety and must be a priority for organized psychiatry. The field of psychiatry must reevaluate its processes of quality assurance to systematically reduce the incidence of these disciplinary actions.
